# Structural differences in the gut microbiome of bats using terrestrial vs. aquatic feeding resources

**DOI:** 10.1186/s12866-023-02836-7

**Published:** 2023-04-01

**Authors:** Alexandra Corduneanu, Alejandra Wu-Chuang, Apolline Maitre, Dasiel Obregon, Attila D. Sándor, Alejandro Cabezas-Cruz

**Affiliations:** 1 Department of Animal Breeding and Animal Production, University of Agricultural Sciences and Veterinary Medicine of Cluj-Napoca, Cluj-Napoca, Romania; 2 Department of Parasitology and Parasitic Diseases, University of Agricultural Sciences and Veterinary Medicine, Cluj-Napoca-Napoca, Romania; 3 UMR BIPAR, Laboratoire de Santé Animale, ANSES, INRAE, Ecole Nationale Vétérinaire d’Alfort, Maisons-Alfort, France; 4 INRAE, UR 0045 Laboratoire de Recherches Sur Le Développement de L’Elevage (SELMET-LRDE), 20250 Corte, France; 5 EA 7310, Laboratoire de Virologie, Université de Corse, Corte, France; 6 School of Environmental Sciences, University of Guelph, Guelph, ON N1G 2W1 Canada; 7 Department of Parasitology and Zoology, University of Veterinary Medicine, Budapest, Hungary; 8 ELKH-ÁTE Climate Change: New Blood-Sucking Parasites and Vector-Borne Pathogens Research Group, Budapest, Hungary

**Keywords:** Bats, Microbiome, Bacterial community assembly, Myotis, Miniopterus

## Abstract

Bat gut microbiomes are adapted to the specific diets of their hosts. Despite diet variation has been associated with differences in bat microbiome diversity, the influence of diet on microbial community assembly have not been fully elucidated. In the present study, we used available data on bat gut microbiome to characterize the microbial community assembly of five selected bat species (i.e., *Miniopterus schreibersii*, *Myotis capaccinii*, *Myotis myotis*, *Myotis pilosus*, and *Myotis vivesi*), using network analysis. These bat species with contrasting habitat and food preferences (i.e., *My. capaccinii* and *My. pilosus* can be piscivorous and/or insectivorous; *Mi. schreibersii* and *My. myotis* are exclusively insectivorous; while *My. vivesi* is a marine predator) offer an invaluable opportunity to test the impact of diet on bat gut microbiome assembly. The results showed that *My. myotis* showed the most complex network, with the highest number of nodes, while *My. vivesi* has the least complex structured microbiome, with lowest number of nodes in its network. No common nodes were observed in the networks of the five bat species, with *My. myotis* possessing the highest number of unique nodes. Only three bat species, *My. myotis*, *My. pilosus* and *My. vivesi*, presented a core microbiome and the distribution of local centrality measures of nodes was different in the five networks. Taxa removal followed by measurement of network connectivity revealed that *My. myotis* had the most robust network, while the network of *My. vivesi* presented the lowest tolerance to taxa removal. Prediction of metabolic pathways using PICRUSt2 revealed that *Mi. schreibersii* had significantly higher functional pathway’s richness compared to the other bat species. Most of predicted pathways (82%, total 435) were shared between all bat species, while *My. capaccinii*, *My. myotis* and *My. vivesi*, but no *Mi. schreibersii* or *My. pilosus*, showed specific pathways. We concluded that despite similar feeding habits, microbial community assembly can differ between bat species. Other factors beyond diet may play a major role in bat microbial community assembly, with host ecology, sociality and overlap in roosts likely providing additional predictors governing gut microbiome of insectivorous bats.

**Supplementary Information:**

The online version contains supplementary material available at 10.1186/s12866-023-02836-7.

## Introduction

Over the past years, research studies have been focusing on understanding the diversity and function of host-associated microbiome, both in humans and animals [1–3]. The gut microbiome consist of microorganism (i.e., bacteria, bacteriophage, fungi, protozoa, and viruses) which play important roles in maintaining the health of an organisms and can influence basic biochemical and physiological processes (e.g., digestion, immune system, metabolic rate) [4, 5]. The composition of the gut microbiome is influenced by different factors, like genetics [6, 7], age [8], environment and habitat [9, 10] or diet [11, 12]. All of these factors can affect the structure of gut microbiome not only in humans, but also in invertebrates or other mammals, such as bats.

Bats are one of the most diverse, complex, and widespread groups of mammals in the world, with more than 1450 species described, which inhabit a very diverse range of habitats due to their ability to fly [13]. Studies on bat microbiome were performed on different types of samples, such as saliva, skin [14–18], tissues [15, 16], urine [17], but the most abundant are on gut [18, 19]. Bats consume a large variety of food (e.g., fruits, blood, insects, fish, nectar) [13] and their gut microbiome is adapted to that specific diet [20, 21].

Most studies on the bat gut microbiome used metagenomic sequencing approach targeting the 16S rRNA gene, especially the V3-V4 region [18, 22, 23]. It was shown that diet in these mammals can have a large influence on the gut host-microbiome diversity. Phillips et al. [24] suggested that bats that feed on blood, insects, nectar, and fruits may have a higher microbiome diversity, whilst Banskar et al. [25] showed that the microbial communities of frugivorous and insectivorous bats are similar. In contrast, Carrillo-Araujo et al. [20], analyzed the gut microbiome of phyllostomid bats and the results showed that nectarivorous and frugivorous diets have low diversity and less specificity compared with bats feeding on blood and insects which had the highest diversity.

Using next-generation sequencing, the results obtained can offer information regarding the taxonomic composition of a specific sample [15, 26], but with the help of bioinformatics, more complex analysis can be performed. Microbial co-occurrence networks are a useful approach to investigate microbial community assembly and their dynamics in different types of organisms [27, 28]. With the analysis of the microbial co-occurrence networks it is possible to identify and predict bacterial associations, and also to identify the host-associated core microbiome which is characteristic of each individual [27]. Besides the characterization of co-occurrence networks and core microbiome, the 16S rRNA data can be used to predict metabolic function, such as pathways and enzymes by matching taxonomic data to metabolic reference databases [29, 30].

In the present study, we used a network analysis approach, based on 16S rRNA gene data published by Aizpurua et al. [31], to characterize the microbial community structure of five selected bat species (i.e., *Miniopterus schreibersii*, *Myotis capaccinii*, *Myotis myotis*, *Myotis pilosus*, and *Myotis vivesi*), with contrasting habitat and food preferences. The characteristic traits of each bat species considered in this study are presented in Table 1. In the original paper, a taxonomic characterization of the gut microbiome of 15 different bat species was also performed, showing differences between bats that have different feeding habits. While most studies performed on bat-gut microbiome are characterizing the presence and relative abundance of different (or selected) bacterial taxa, the aims of our study were to: (i) represent and characterize the microbial co-occurrence networks, (ii) identify of core microbiomes, and (iii) predict the metabolic functions, such as pathways in order to evaluate the importance of the hosts’ life-history characteristics on the microbiome constitution.

**Table 1:** Bat species traits

	***Mi. schreibersii*** (*n* = 10)	***My. capaccinii*** (*n* = 22)	***My. myotis*** (*n* = 9)	***My. pilosus*** (*n* = 13)	***My. vivesi*** (*n* = 8)
**Roosting**					
Summer roost	Cave	Cave	Cave	Cave	Crevice
Roost size (individuals)	1000–10,000	100–1000	100–1000	1000–10,000	1–8
Sexes roosting	Together	Separately	Separately	Separately	Separately
Roost-types	Multispecies, multispecies mixed clusters	Multispecies, multispecies mixed clusters	Multispecies, clustering in single species groups	Multispecies, Multispecies, clustering in single species groups	Single species
Roosting frequently together with	*My. capaccinii, My. myotis*	*Mi. schreibersii, My. myotis*	*Mi. schreibersii, My. capaccinii*	others	-
Hibernating	Yes	Yes	Yes	Yes	No
Hibernation roost	Cave	Cave	Cave	Cave	-
Roost size (individuals)	1000–10,000	10–50	10–100	1000–10,000	
Sexes hibernating	Together, same cluster	Together, clusters are formed from the same sex individuals	Together, small clusters	Together, small cluster	-
Hibernating frequently together with	*My. capaccinii*	*Mi. schreibersii*	-	Others	-
**Morphology**					
FA length (mm)	43–47.1	38.1–44	55–66	52.1–63.5	53.7–63
Weight (g)	10–14	7–10	20–27	11.7–32.5	22–28
**Feeding and habitat**					
Food	Small to medium sized flying Lepidotera (70–90%), Neuroptera, Diptera, Trochoptera and Coleoptera	Small, flying arthropods above water: flies, caddisflies, small Diptera, Hymenoptera, occasionally small fish	Large arthropods: ground-dwelling, large Coleoptera (Carabidae), Orthoptera and Arachnida	Fish (30%) and arthropods (70%), Coleoptera, Diptera, Trichoptera, Lepidoptera	Marine fish and crustaceans
Feeding specialization	Aerial hawker	Trawling on slow-flowing water surface	Ground gleaning, slow overflight	Trawling on slow-flowing water surface	Trawling in marine lagoons
Habitat	Terrestrial	Freshwater	Terrestrial	Freshwater	Marine
Hunting habitat	Above and inside forests and grasslands, orchards and parks	Wetlands, with open water surface, either lakes or rivers	Forests and grasslands, needs clearings inside forest	Wetlands, dependent on slow flowing rivers and lakes	Marine lagoons
Home range (km)	3–15	20	26	?	?
Hunting speed	Fast	Medium	Slow	Fast	Slow
Hunting height (m)	3–20	0.2–0.5	0.3–0.7	0.3–0.7	0.2–0.5
**Reproduction**					
Mating	Swarming/autumn (September–October)	Swarming/autumn (September–October)	Swarming/autumn (September–October)	Autumn to winter (September-April)	Summer to autumn (July–September)
Gestation type	Immediate fertilization, delayed implantation	Sperm stored until ovulation (after hibernation)	Sperm stored until ovulation (after hibernation)	Sperm stored until ovulation (after hibernation)	Sperm likely stored
Sociality	Highly social	Social only inside nursing colonies, small clusters otherwise	Social only inside nursing colonies, males single	Social only inside nursing colonies, males single and highly territorial	Territorial
**Range and movements**					
Geographic range	Palearctic, Mediterranean + Middle East	Palearctic, Mediterranean + Middle East	Palearctic, European endemic	Oriental, SE China, N Vietnam and Laos	Nearctic, Baja California
Extent of occurrence (km^2^)	19.946.710	5.387.022	7.071.111	1.796.095	134.00
Migration	Medium distance			medium distance	
Seasonal movements (km)	100–400	10–50	50–100	100–400	100–400
**Lifespan**					
Generation length (year)	5.5	6	7.8	5	7
Max longevity (year)	16	?	22	?	?

## Materials and methods

### Original data set

For data analysis, we used a previously published set of 16S rRNA gene sequencing data. The original study described the role of gut microbiome in the dietary niche of different bat species [31]. Insectivorous and piscivorous bat species were considered in that study and the taxonomic and functional characteristics of the gut microbiome were analyzed. Their results showed that the gut microbiome of piscivorous bat species is different from the gut microbiome of insectivorous ones. Regarding the microbial community, the highest similarities were observed between two piscivorous bat species: *My. capaccinii* and *My. pilosus* with different dominant bacteria influenced by habitat (Mediterranean/temperate-subtropical) and *My. vivesi,* which showed different microbial communities. Data sets were generated targeting the V3 and V4 hypervariable regions of the 16S rRNA gene using the pairs of primers 341F/806R followed by sequencing on an Illumina MiSeq platform. The raw sequence data are available in the EMBL-EBI repository under the project accession number PRJEB47836.

### Analysis of 16S rRNA sequencing dataset

The 16S rRNA gene sequences used in this study were downloaded in fastq format from EMBL-EBI repository. The DADA2 software [32] implemented in QIIME2 was used for demultiplexing the 16S rRNA gene sequences and quality trimming based on the average quality per base of the forward and reverse reads. The first 22 nucleotides were removed and then the total length was trimmed to 465 base pairs in both forward and reverse reads. Both reads were merged, and chimeric variants were removed. The resulting sequences were taxonomically assigned applying a pre-trained naive Bayes taxonomic classifier [33], based on SILVA database version 132 [34], and the primers used in the original dataset (341F/806R). The taxonomic data table obtained was collapsed at genus level and taxa that had less than 10 total reads from each set were removed. The resulted amplicon variant sequences (ASVs) table was used for calculation of Jaccard coefficient of similarity, network analysis, identification of the core microbiome, and prediction of the functional traits. The beta diversity between samples of each bat species was compared using the Jaccard coefficient of similarity and Jaccard clusterization analysis was conducted using Vegan implemented on R studio (RStudio 2020).

### Co-occurrence networks, identification of core microbiome and network resistance analysis

The analysis of the co-occurrence networks was performed using the Sparse Correlations for Compositional Data (SparCC) method [35] implemented in R studio (RStudio, the script code used can be found in Supplementary File 1). To calculate the correlation matrix, taxonomic ASVs tables were used, selecting correlation coefficients with a magnitude larger or smaller than 0.6 (for the weight of the interactions). The Gephi 0.9.2 [36] software was used for visualization of all networks (i.e., with equal-size nodes, and those with node size proportional to eigenvector centrality (EV), or betweenness centrality (BNC)) and the calculation of the topological features and taxa connectedness (i.e., number of nodes and edges, modularity, network diameter, average degree, weighted degree, clustering coefficient, and centrality metrics). We used the online available Venn diagram tool (http://bioinformatics.psb.ugent.be/webtools/Venn/) for visualizing the number of nodes and shared taxa between networks. Also, Venn diagrams were used to visualize the central nodes for the two parameters, EV and BNC.

To characterize the taxonomic core microbiome of all bat species included in this study the SparCC method [35] implemented in R studio (RStudio 2020) with a threshold of 0.9 and -0.9 for co-occurrence correlation was used. The Gephi 0.9.2 software was used for network visualization for those species that had a core.

The robustness of the co-occurrence networks was determined using an attack tolerance test with the package NetSwan for R [37]. For this analysis all networks were subjected to the systematic removal of nodes, using three different types of attacks: (i) random with 100 iterations, (ii) direct where nodes are removed in decreasing order of their BNC value, and (iii) cascading where BNC values are recalculated after each node removed. Also, the loss of connectivity was assessed for each method.

### Differential network analysis

Using the package ‘NetCoMi’ [38] in R studio a comparison of the similarity of the most central nodes between two networks was performed. The result of this comparison is a Jaccard index, for each of four local centrality measures (i. e. degree, BNC, closeness centrality, EV) for the nodes, as well as for those sets of hub-nodes for the two networks compared. The Jaccard index of 0 shows complete dissimilarity between the parameters, while the value 1 indicates the highest similarity between these network parameters [38].

### Prediction of the functional traits in gut the microbiome of bats

We used PICRUSt2 to predict the functional profile of bacterial communities based on the 16S rRNA gene sequences [39]. The amplicon sequence variants (ASVs) were inserted into a reference tree (with a NSTI cut-off value of 2), to obtain gene family copy numbers of each ASV. The Kyoto Encyclopedia of Genes and Genomes (KEGG) orthologs (KO) [40], Enzyme Classification numbers (EC), and Cluster of Orthologus genes (COGs) [41] were used for the functional annotations of the pathways. Pathway profiles were mapped based on the MetaCyc database [42], highlighting both the shared as well as the unique pathways using a Venn diagram. The MetaCyc pathway was calculated based on the abundance of the predicted EC numbers. Taxa contribution to pathways was inferred using data obtained from the pathways analysis and set the threshold at 10% contribution for each taxon to a specific pathway (only for three bat species).

### Statistical analyses

To test the similarity of the most central nodes, we calculated two *p*-values. *p*(J ≤ j) and *p*(J ≥ j) for each Jaccard’s index, representing the probability that the observed value of Jaccard’s index is “less than or equal” or “higher than or equal”, respectively, to the Jaccard value expected at random. Differences were considered significant for *p*-value < 0.05. An ANOVA test with post hoc analysis was performed in GraphPad Prism 9 to highlight the statistical differences for all three different types of attacks used for testing network robustness. The alpha diversity was determined using the observed features index. To test for differences in alpha diversity metrics between groups, as well as for comparing differential pathways abundances we used Kruskal–Wallis one-way ANOVA-s.

## Results

### Assembly of bat microbiome

**Fig. 1:**
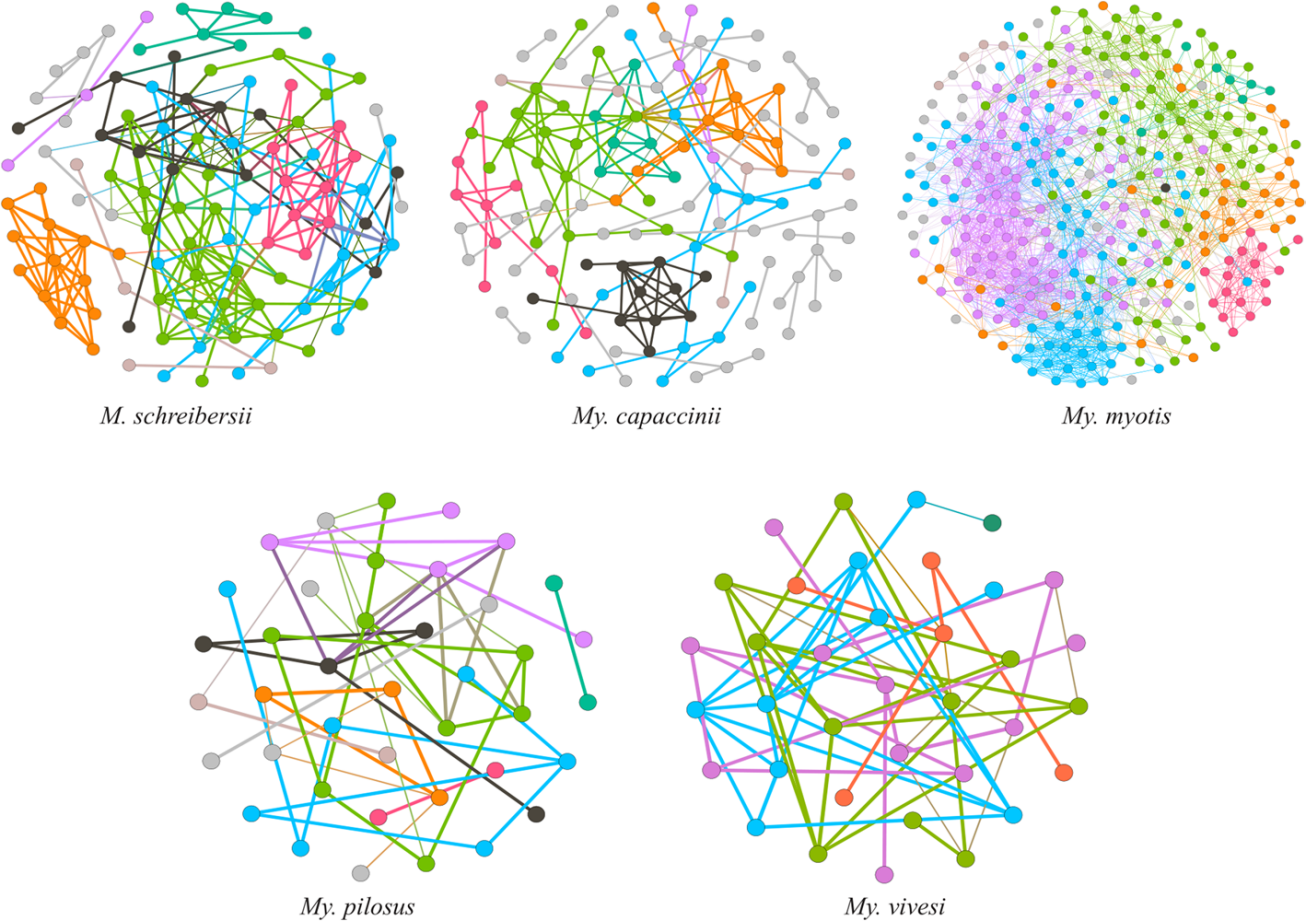
Microbial co-occurrence networks of different bat species. The bacterial co-occurrence networks were constructed based on 16S rRNA gene sequences obtained from a previous study (Aizpurua et al., 2021). Nodes represent bacterial taxa and edges represent co-occurrence correlation. The node color is based on the modularity class. Thus, nodes with the same color belong to the same cluster. The edges are connecting links with negative and positive interactions, respectively (SparCC > 0.60 or < -0.60). Only nodes with at least one connecting edge are shown

**Fig. 2:**
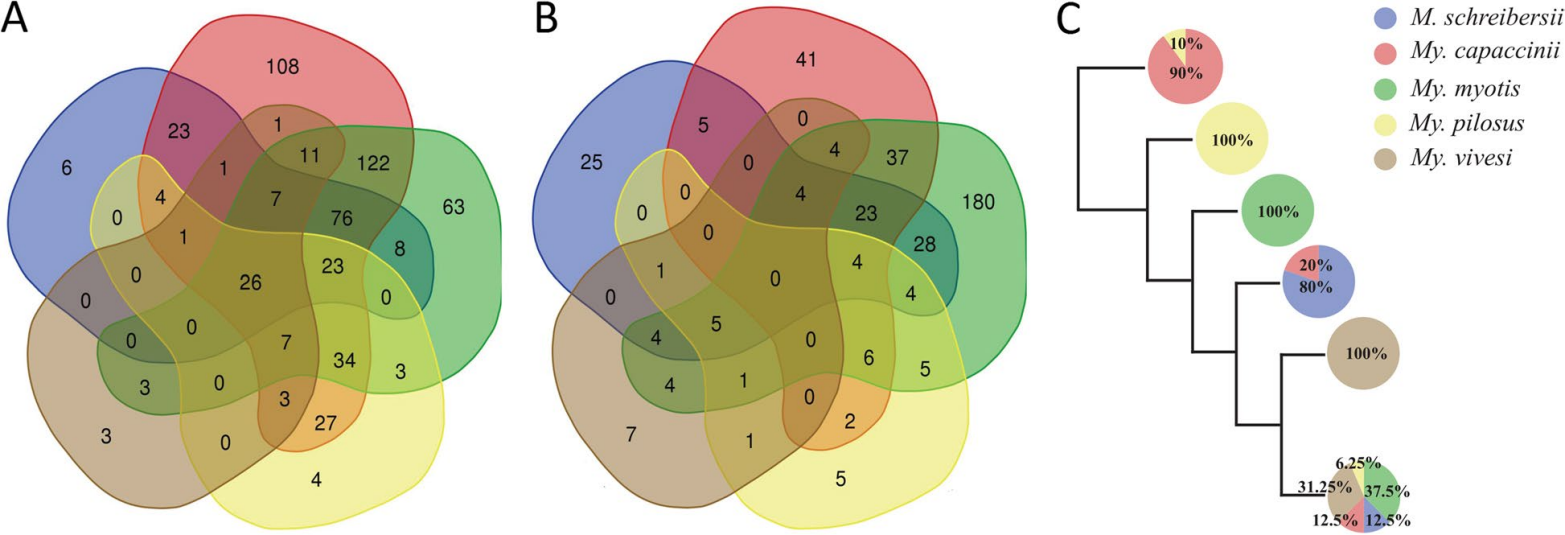
Cladogram and Venn diagrams of taxa and nodes. The Venn diagrams are showing the number of bacterial taxa in the microbiome (**A**) and nodes in the network (**B**) that are common or unique between all five bat species. The cladogram (**C**) displays the simplified Jaccard clusterization of bat microbiome from the samples analysed. Percentual values represent proportion of samples of each bat species in each defined cluster

The bacterial community structure was determined by inferring co-occurrence networks in five different bat species (Fig. 1, Table 2). The obtained networks show that each bat species has distinct patterns of bacterial co-occurrence networks and there are major differences between all bat species in the number and identity of bacteria present in the gut microbiome (Fig. 2A, Supplementary Table 1). The topological features show the highest number of nodes and edges for *My. myotis* and the lowest number of nodes for *My. vivesi*, whilst the lowest number of edges is observed for *My. pilosus* (Table 2). *My. capaccinii* shows the highest number of unique taxa (*n* = 108), in contrast to *My. vivesi,* which has the lowest number of unique taxa (*n* = 3). A total of 26 taxa are shared between all five bat species. Regarding the number of shared nodes between all bat species networks, the Venn diagram shows no common nodes (Fig. 2B, Supplementary Table 2), with *My. myotis* having the highest number of unique nodes (*n* = 180). The networks show that there are differences in the assembly of the bacterial community. The Jaccard's coefficient of similarity of samples in the fives species show that most of the samples can be grouped in a different cluster by species. The Jaccard clusterization and network analysis highlights the fact that both the beta diversity and assembly of the gut microbiome of the five considered species is different (Fig. 2C).

**Table 2:** Topological features of the microbial co-occurrence networks

Topological parameters	Bat species				
	*Mi. schreibersii*	*My. capaccinii*	*My. myotis*	*My. pilosus*	*My. vivesi*
Nodes	107	128	317	40	36
Edges	239	173	2136	51	57
Positive	207 (86.62%)	167 (96.53%)	1461 (68.39%)	46 (78.26%)	54 (85.18%)
Negative	32 (13.38%)	6 (3.46%)	675 (31.60%)	10 (21.73%)	8 (14.81%)
Network diameter	12	15	7	6	8
Average degree	2.731	0.73	13.476	0.773	1.81
Weighted degree	1.358	0.44	3.723	0.312	0.894
Average path length	4.62	5.018	3.234	2.736	3.346
Modularity	0.898	0.868	1.341	0.867	0.872
Number of modules	14	27	27	13	5
Average clustering coefficient	0.459	0.523	0.538	0.465	0.465

**Fig. 3:**
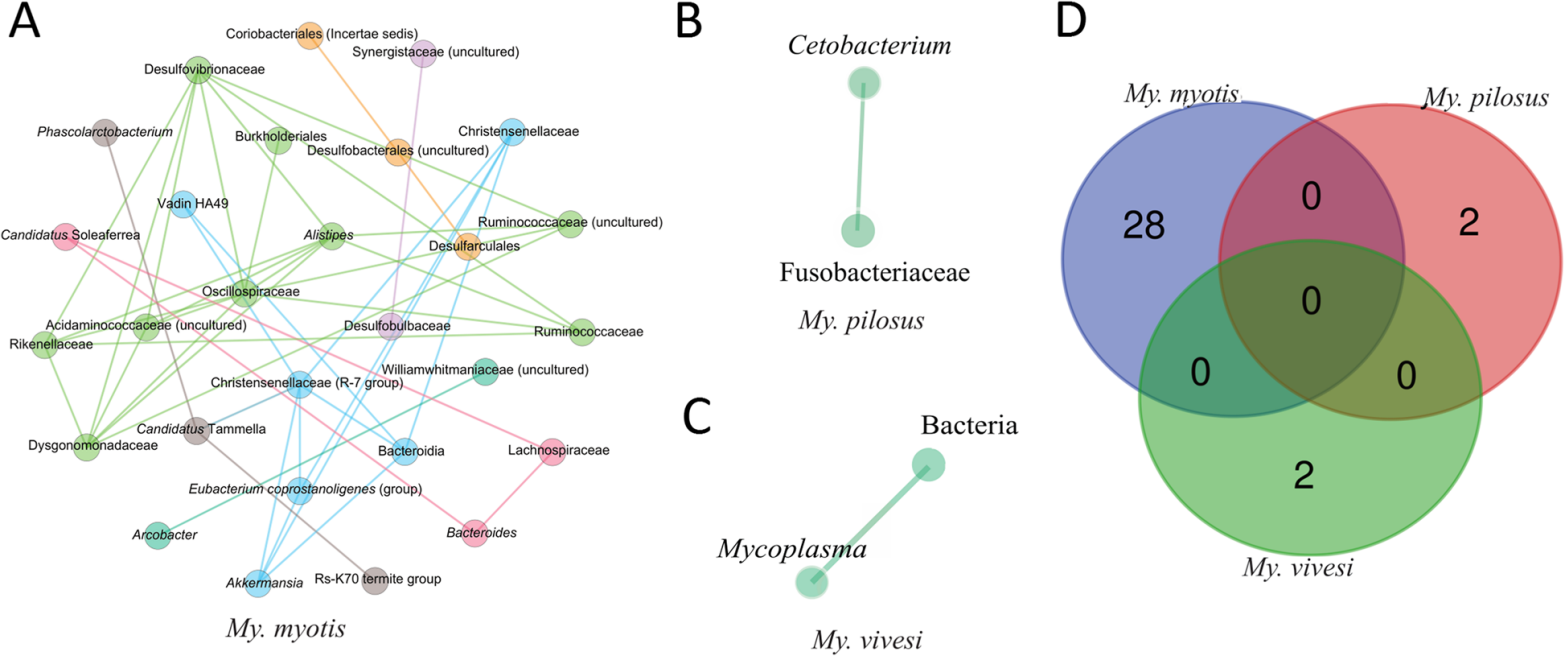
Core co-occurrence networks of three bat species. Co-occurrence networks of *My. myotis* (**A**), *My. pilosus* (**B**) and *My. vivesi* (**C**) are shown. Nodes correspond to bacterial taxa, and only those with at least one significant correlation are represented. The color of the nodes is based on the modularity class. All edges with positive and negative correlation are represented (SparCC > 0.90 or < -0.90). The Venn diagram (**D**) is showing the number of core bacteria that are common or unique among the three bat species

The bat-associated core microbiome was analyzed to determine the particularities for each species (Fig. 3). Only three bat species presented a core microbiome within the selected threshold (SparCC > 0.9): *My. myotis* (Fig. 3A), *My. pilosus* (Fig. 3B) and *My. vivesi* (Fig. 3C), with the most abundant being present in *My. myotis*. Core networks were characterized by the presence of different taxa in each species and none of these bacterial taxa were shared between *My. myotis*, *My. pilosus* and *My. vivesi* (Fig. 3D). The core network of *My. myotis* showed the presence of different bacteria (e.g., *Akkermansia*, *Arcobacte* Burkholderiales, Coriobacteriales**,** *Fusobacteriaceae, Synergistaceae*). In both cases of *My. pilosus* and *My. vivesi*, only two taxa were present in the core microbiome (Fig. 3B, C). These results show that *My. myotis* has the highest structural complexity of the gut microbiome community, while both *My. pilosus* and *My. vivesi* show a more specialized, but less diverse structure since all nodes are unique and specific for each bat species.

### Node centrality distribution and network robustness

**Fig. 4:**
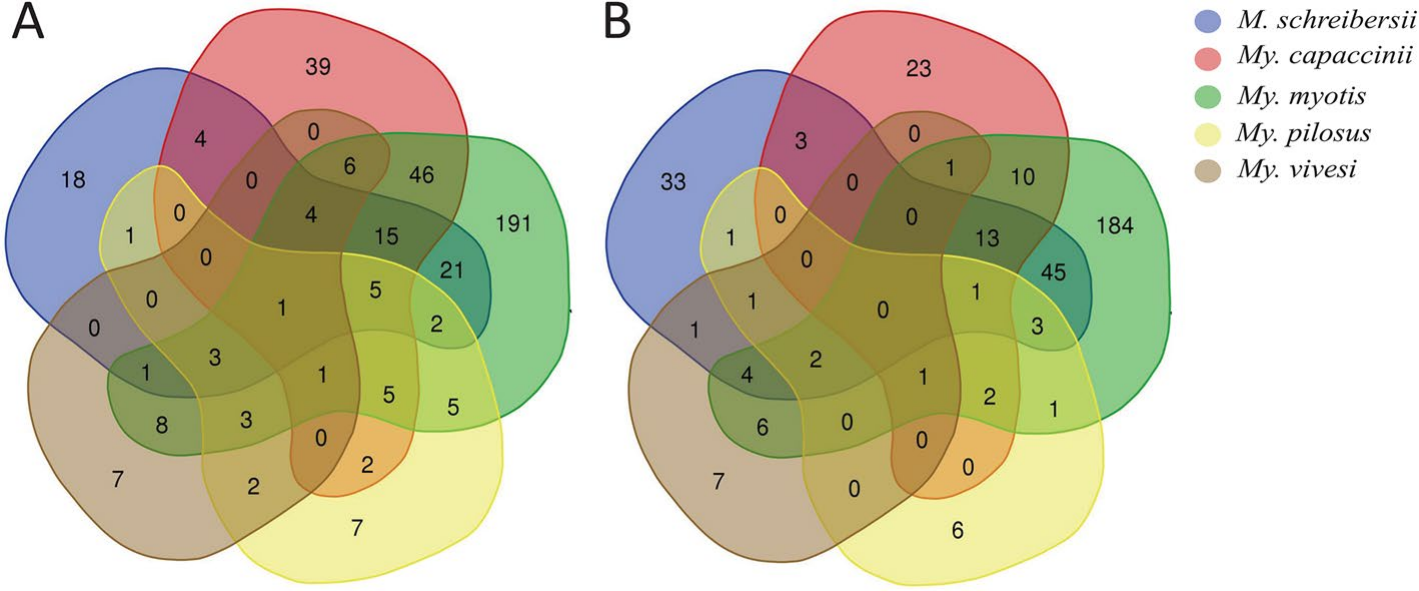
Venn diagrams of microbial co-occurrence networks of different bat species. The Venn diagrams are showing the number of central nodes using two different parameters: eigenvector (**A**) and betweenness centrality (**B**) that are common or unique between all five bat species

Co-occurrence networks comparisons using the EV and BNC parameters highlight once more the complexity of *My. myotis* network, while *My. pilosus* had the lowest complexity. The size of the nodes represents their influence in a particular network, depending on each parameter (Supplementary Fig. 1A, B). Venn diagrams from the Fig. 4A, B represent the number of the central nodes shared and unique in each species for EV (Supplementary Table 3) and BNC (Supplementary Table 4) parameters. There is one shared node between all bat species when EV parameter is used. Also, it is shown a higher number of nodes for the bat species *My. myotis* for both EV and BNC. The observed Jaccard’s index for local centrality measures (i.e., degree, BNC, closeness centrality, eigenvector centrality, and hub taxa) was significantly lower than expected by random for all the comparisons (Supplementary Table 5). The Jaccard index showed statistically significant differences for all network parameters between each pair of comparisons of the five bat species, suggesting differences in network centrality distribution for each network (Supplementary Table 5).

**Fig. 5:**
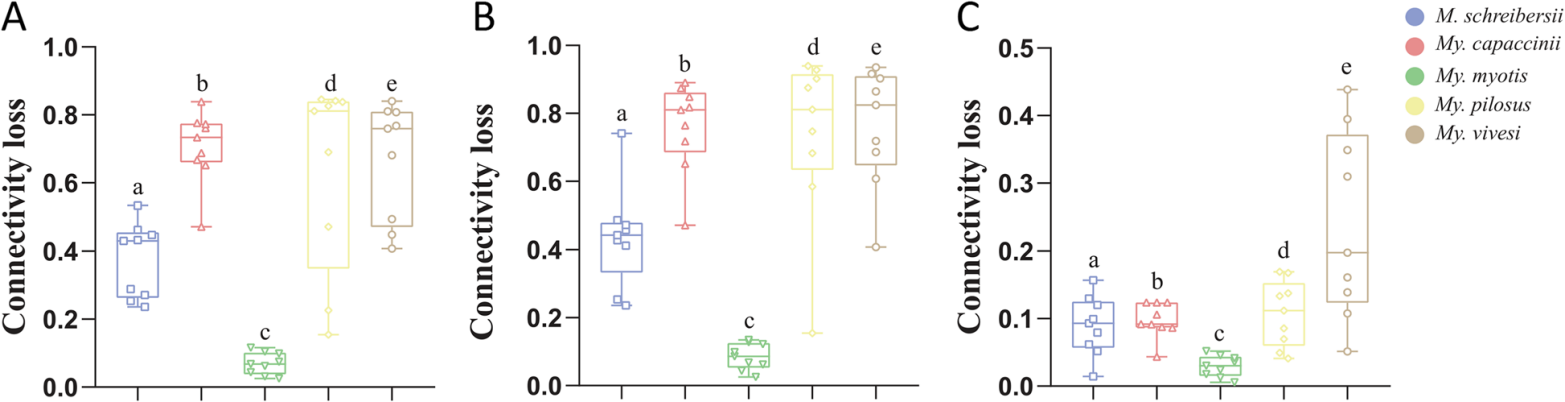
Comparison of network tolerance to taxa removal based on three different types of attack. The resistance of the networks for the microbiome of five different bat species was measured by the removal of nodes using three different attacks: direct (**A**), cascading (**B**), or random (**C**). Nodes removal was based on their BNC value. All pairwise comparisons were statistically significant. Loss of connectivity values ranges between 0 (maximum of connectivity between nodes) and 1 (total disconnection between nodes). The statistical analysis used was an ANOVA test with post hoc analysis

To determine the robustness of the networks, we tested their tolerance to different types of attack: using direct (Fig. 5A), cascading (Fig. 5B), and random nodes (Fig. 5C) removal. Similar results were reached in the networks by all three approaches of taxa removal. Statistical analysis was performed using the ANOVA test, showing that the bat species *My. myotis* has the highest ‘robustness’ (i.e., lowest susceptibility to network attack). For the same bat species less than 20% of network connectivity is lost after removal of around 50% of the nodes using each type of attack. By comparing the loss of connectivity, measured by random attacks, of the networks of all five bat species, the network of *My. vivesi* presents the lowest tolerance to taxa removal (Supplementary Fig. 2). Random taxa removal result in the lowest loss of connectivity in each network, while cascading attacks induce the lowest tolerance in all networks (Supplementary Fig. 3).

### Predicted functional profiles associated to bat microbiomes

**Fig. 6:**
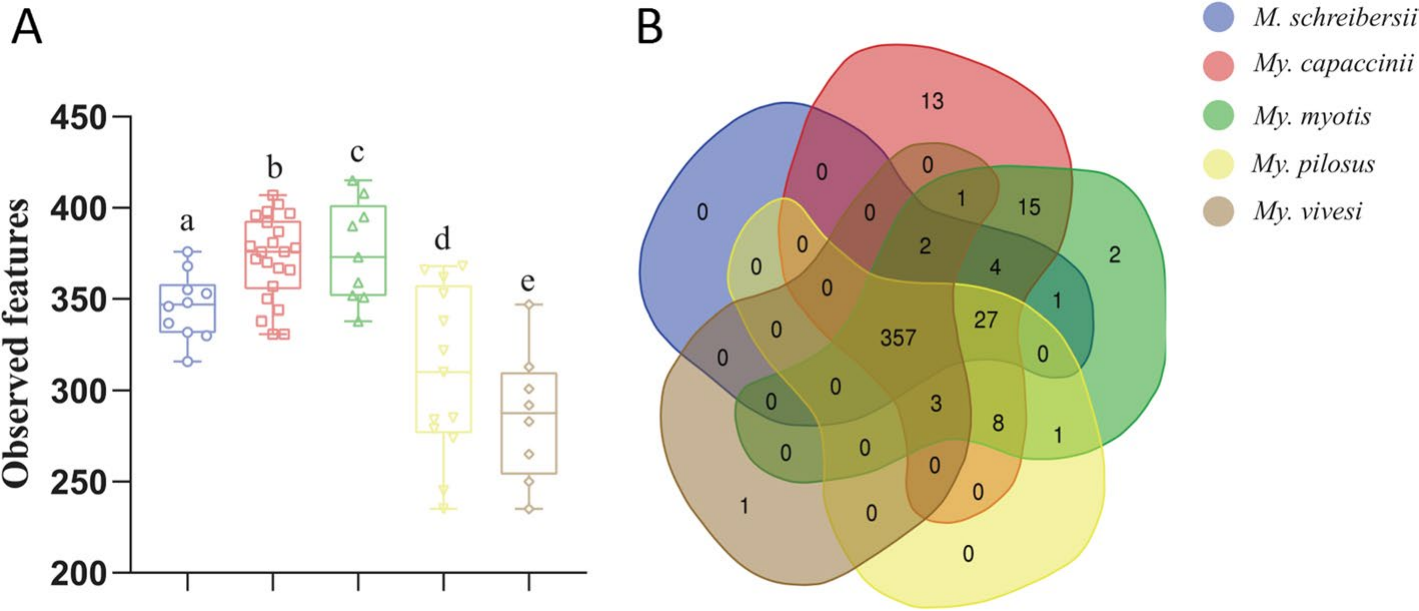
Alpha diversity and Venn diagram of pathways. The comparison of alpha-diversity with the Observed features index for all bat species together with the statistical analysis is represented (**A**). All pairwise comparisons were statistically significant. Details regarding the p-value for the index can be found in Table 3. The Venn diagram (**B**) is showing the comparison of unique and shared pathways present in the bat species considered in this study

For each bat species, the analysis of alpha diversity of the observed features (number of pathways) was performed (Fig. 6A). The analysis of alpha diversity of functional pathways showed that *Mi. schreibersii* have lower richness functional pathways than *My. capaccinii* and *My myotis* (Kruskal–Wallis, *p* < 0.05, Fig. 6A).

**Table 3:** Statistics for the observed feature index comparisons for pathways

Group 1	Group 2	H	*p*-value
*Mi. schreibersii* (*n* = 10)	*My. capaccinii* (*n* = 22)	7.87	**0.005008**
	*My.myotis* (*n* = 9)	5.22	**0.022243**
	*My. pilosus* (*n* = 13)	3.23	0.071957
	*My. vivesi* (*n* = 8)	9.67	**0.001872**
*My. capaccinii* (*n* = 22)	*My.myotis* (*n* = 9)	0.08	**0.001872**
	*My. pilosus* (*n* = 13)	15.42	0.777206
	*My. vivesi* (*n* = 8)	15.52	**0.000086**
*My. myotis* (*n* = 9)	*My. pilosus* (*n* = 13)	8.24	**0.004075**
	*My. vivesi* (*n* = 8)	11.34	**0.000757**
*My. pilosus* (*n* = 13)	*My. vivesi* (*n* = 8)	1.26	0.261486

The analysis of the identity of the different predicted pathways showed that 82% (357, total 435) of the pathways were shared between all bat species. Moreover, *My. capaccinii*, *My. myotis* and *My. vivesi* showed unique pathways (Fig. 6B**,** Supplementary Table 6). Indeed, *My. capaccinii* showed the highest number of unique pathways (i.e., 13), while *Mi. schreibersii* and *My. pilosus* lacked unique pathways (Fig. 6B). Differential abundance analysis showed that several predicted pathways are different between different bat species (Supplementary Fig. 4). The analysis of the specific pathways revealed the presence of three ‘superpathways’ for *My. capaccinii* (i.e., fermentation (PWY-7401), pyrimidine nucleobase/ribonucleoside degradation (PWY-7209) and quinolone and alkyquinolone biosynthesis (PWY-6662)), one for *My. vivesi* (proteinogenic amino acid biosynthesis (PWY-7528)). *My. myotis* had pathways involved in both degradation (aromatic compound degradation – PWY-7002) and biosynthesis (antibiotic biosynthesis – PWY-6919). These results suggest that the constituents of *My. capaccinii*’s gut microbiome, a piscivorous bat, have extensive functional roles, highly differing from the ones of the other four bat species analyzed.

**Fig. 7:**
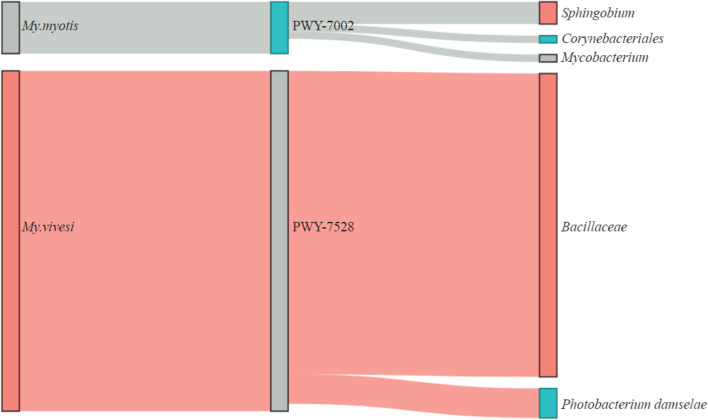
Contribution of commensal bacteria to pathways in the microbiome from two bat species. The alluvial plot represents the presence of specific pathways for two different bat species. The bacterial taxa contributing to those specific pathways are also represented. Node segments by columns are showing the host (first column), pathways (second column), and bacterial taxa (third column). The size of the node is proportional to the abundance of contributing host, pathway, or bacterial taxa. The cords represent the connection between the host, pathways, and taxa. The contribution of each taxon to different pathways is proportionally represented by the size of the cords

For the same three bat species, an analysis regarding the taxa contribution to those specific pathways was performed. Only taxa with the highest abundance and contribution (more than 10%) were selected. The bat species *My. capacinii* and one specific pathway for *My. myotis* were eliminated from the analysis because all the taxa contributing to those 14 specific pathways had very low values. For *My. myotis*, only three taxa were selected for the contribution to the pathway PWY-7002, *Sphingobium* having the highest contribution, followed by *Corynebacterriales* and *Mycobacterium* genus. In particular, for *My. vivesi* the taxa with the highest contribution were represented by *Bacillaceae*, followed by *Photobacterium damselae* (Fig. 7).

## Discussion

As the number of studies on the composition and the role of gut microbiome had increased in the past years, more and more animals were analyzed. Due to their role in the epidemiology and spreading of diseases, the gut microbiome of bats was also a subject of great importance. In the present study, we used available published data [31] to perform a network analysis of the gut bacterial community for five different bat species. We considered the diet as the most important factor that can influence the microbiome. It was showed that microbiome and diet plays an important role in human health and can influence the appearance of diseases: diabetes, autoimmune diseases, inflammatory bowel diseases, and some types of cancer [43–46]. Animal-based diet has a greater impact on the gut microbiome than a plant-based diet as it was shown both in humans and in bats [20, 47]. Human diets show wide variances around the world (availability and type of food, urbanization, lifestyle) and has an important impact on their health, both in children and adults, highlighting the need for balanced food [48]. Bats are conservative in their diet choice, with high similarity within the same dietary group (insectivorous, nectarivorous, frugivorous), and it is rare to include nonspecific food resources. An example is the group of frugivorous bats that rarely eat insects, just doing so in order to balance their nutrients [49].

Bats’ diet plays a major role in building the gut microbiome [20, 21, 24]. Phyllostomid bats are the most varied and diverse family of bats (over 190 species), with most species being insectivorous, but there are also sanguivorous, carnivorous, nectarivorous, and frugivorous species in the group. A study on the gut microbiome of different species from this family showed that those that feed with blood and insects had the most diverse microbiome and a more clustered arrangement of bacterial community compared to those that have a plant-based diet [20]. In contrast, the study performed by Phillips et al., [24], suggested that the microbiome diversity is higher in bats relying on a plant-based food-source compared to bats using animal-based nutrients. Still, the presence of shared bacterial taxa observed between frugivorous and insectivorous bats studied in India, suggests an overlap in their diet [25].

The microbiome analysis performed in this study included bats from two different families (Vespertilionidae and Miniopteridae) that predominantly feed on insects, thus sharing a similar fundamental resource. Similarly to other studies [19], we found no major proof of the effect of host phylogeny per se, none of the characteristics of the individual microbiomes mirrored host phylogeny (Fig. 2, also), so we looked for further physiological predictors. Diet was shown as very important building factor of microbiome structures and one may expect the major differences between constituent microbiomes either along the diversity of their diet components (few, basically similar species consumed in large quantities vs. diverse array of taxonomically different groups) or basic position in the trophic pyramid (ei. consumption of chiefly primary consumers – insect herbivores or predatory insects). The co-occurrence analysis of the networks showed that *My. myotis* had not only the highest microbial diversity, but also the highest number of nodes and edges resulting in higher network complexity, while the two most distant from *My. myotis* (both in terms of phylogeny as well geographically) were positioned *My. vivesi* and *My. pilosus,* having the lowest values regarding the number of nodes and edges, as well a more specialized microbiome (Fig. 1, 3, Table 2.). This may be partially caused the contrast between the high diversity of prey items in case of *My. myotis* (Table 1, diverse assemblages of ground-dwelling, terrestrial groups of Coleoptera, Othoptera and Arachinda, but also Miriapoda, Heteroptera or Lepidoptera, with up to 40–60 different species—see also [50, 51]), in contrast to the purely marine sourced, arthropod (Crustacea) and fish consumer *My. vivesi* (up to 70% food made by Crustacea, [52]), or the fresh water-fish specialist *My. pilosus* (up to 60% food made by 3 fish species, see [53–55]).

Microbiomes of both *Mi. schreibersii* (primarily a terrestrial Lepidoptera and Diptera preying species, [56, 57]), as well *My. capaccinii* (primarily a low diversity, freshwater Diptera-specialist, [58, 59]) showed more similarity to each other, or to *My. myotis* (Table 2, Figs. 2 and 4, Supplementary Figs. 1, 2 and 3), then to the other two bat species with a similarly narrow, specialized diet (*My. vivesi* and *My. pilosus*). This may be caused by other factors than purely diet diversity. While *Mi. schreibersii* may share similar habitats with *My. myotis* (thus theoretically they may hunt the same primary consumers – Lepidoptera and some Diptera)*,* the overlap between their food palette is non-significant, due to the strikingly different hunting technique and prey-size (Table 1, see also [51, 56, 60]). The differences between the feeding regimes of *Mi. schreibersii* and *My. capaccinii*, or *My. myotis* and *My. capaccinii*, are even more significant, as the latter is a really narrow specialist, relying primarily on small-sized aquatic Diptera (chiefly Chironomidae) and Trichoptera [58, 59], thus fully avoiding not only terrestrial hunting grounds, but also most terrestrial insects. Also, *My. capaccinii* does not showed the overlap in microbiome constituents or structure with the two other aquatic feeders species (*My. vivesi* and *My. pilosus*), although their consumption of aquatic primary consumers was similar (with *My. pilosus*, even sharing some Trichoptera and Chironomidae groups/species, see [55, 59]). These three bat species not only showed similarly low levels of taxonomic diversity in the diet, or the avoidance of secondary consumer insects, but also each of them regularly consume fish (vertebrates), so they have the most similar diet. Still, the structure and constituency of their respective microbiome is basically different. In consequence, we suspect that hosts’ diet is not the single and most important source or predictor for gut microbiome. We suggest that geographical co-occurrence and physical contacts via shared roosting may have some importance, too. Although the selected species’ ranges are distributed over three continents, there are several species, which show overlapping range, thus they may get into physical contact, favouring in this way easy microbial exchanges. Three of the targeted species may regularly occur even in the same physical space (*Mi. schreibersii*, *My. capaccinii* and *My. myotis,* Table 1, [61]) thus accidental or desired (communal roosting for thermal confort) physical contacts may be common. Especially in the case of *Mi. schreibersii* and *My. capaccinii* this may be important, as these two species are regularly observed to roost in close contact, where individuals may engage in aggressive interactions, considerably easing microbial exchange trough oral contacts [23, 62]. The likely lack of any interaction with individuals of other species, and also the reduced chances of intraspecific interactions in the case of *My. vivesi* (highly territorial, crevice-dwelling species roosting in small groups in contrast to all the other species, which are cave-dwelling and roosting in close-tight, large groups, see Table 1) may be the key not only for the low levels of overlap in microbiome constituents with other bat species (Figs. 1 and 2, Supplementary Fig. 4), but also for the low levels of structuring (Figs. 5 and 6) and extreme fragility of its microbiome (see Fig. 5, Supplementary Figs. 2 and 3). At the beginning of a study, the appropriate sample size that should be used can be calculated using statistical software, but also with some limitations (correlation with the hypothesis, ethics, possibility of sampling) [63, 64]. The different size of the sample can influence result: if a small number of samples are used, this can lead to false premises and loss of money, or on the contrary, if a larger sample is used, this can cause ethical problems, loss of money and time [65, 66]. A bias and limitation of our study is the lack of a sample size calculation. This was not possible because we used already published data and selected only those sequences that were suitable for data analysis.

Overall, the predominant bacterial phyla in the microbiome are gram-positive (Firmicutes) and gram-negative (Bacteroidetes). Studies on human gut microbiome revealed that is richer in the Phyla Firmicutes and Bacteroidetes, while when is higher in Proteobacteria, could be associated with diseases [67]. In contrast, in the analysis of the bats microbial community, the Phylum Proteobacteria was more abundant, followed by Firmicutes [20, 25].

In humans, a diet based on animal food had a great impact on the relative abundance of bacterial taxonomic groups, increasing especially in the microorganisms *Alistipes*, *Biolophila*, and *Bacteroides* and decreasing in those from the phyla Firmicutes [47]. The core microbiome of the bat species *My. myotis* showed especially the presence of gram-negative bacteria (e.g., *Akkermansia*, *Alistipes, Bacteroides, Burkholderiales,* and *Synergistaceae*), associated with different physiological changes (anti-inflammatory effects, activation of CD4 T cells, regulation of homeostasis of oxalic acid) [68–71]. For *My. pilosus* two bacteria were identified, both from the family Fusobacteriaceae, which are gram-negative bacteria that can be associated with diseases [72], and this family was reported to be abundant in patients having non-alcoholic steatohepatitis [73]. For the species *My. vivesi* the bacteria *Mycoplasma* was identified, with an unknown role in their health status or if they can transmit it further to animals and humans [74]. Previous studies also reported the presence of *Mycoplasma* sp. in the gut of *Cynopterus* sp. [25]. The analysis of the core microbiome of frugivorous and insectivorous bats showed that they can share the following bacteria in more than 70% of the samples analyzed: *Deinococcus, Methylobacterium, Sphingomonas, Phenylobacterium, Hymenobacter* [25].

Rarefaction analysis of the observed bacterial species of insectivorous compared with frugivorous bats from India showed that the overall diversity of the gut microbiome is higher for the first ones [25]. For some species from the Phyllostomidae family, the bacterial alpha diversity was higher in bats that feed on blood and insects compared with those that feed on fruits and nectar [20]. In contrast, in our study, we analyse the alpha diversity of functional pathways which showed that the individuals from the Miniopteridae family are richer in functional pathways compared with those from the Vespertilionidae family. This may be caused by the high levels of sociality among species belonging to Miniopteridae [75].

Tools such as PICRUSt, PICRUSt2, Tax4Fun and FaproTax have been developed to infer microbial functional genes from amplicon sequencing data [76]. Studies comparing some of the methods (i.e., PICRUSt, PICRUSt2, and Tax4Fun) revealed that no method was superior to another [76], and PICRUSt2 has been applied on samples from various animals, including arthropods (e.g., *Ixodes* spp.[77], nematodes (e.g., *Caenorhabditis elegans*, [78]), birds (e.g., *Serinus canaria domestica* [79]), and mammals (e.g., humans [39], goats [80], and bats [21], among others. In our study, analysis of the functional prediction for multiple bat species, which are taxonomically different and have distinct feeding strategies, showed that frugivorous bats are very different from insectivorous, carnivores, or blood-feeders. When comparing the animal-based diet with the plant-based diet resulted that 37 functional pathways were differentially abundant between them. For bats that have an animal diet, the most common pathways were associated with biosynthesis and generation of precursor metabolites [21]. In particular, the pathways analyzed in the present paper, especially for three bat species (*My. capaccinii*, *My. myotis* and *My. vivesi*) are represented by biosynthesis and degradation (Supplementary Table 2). The highest number of particular pathways were present for the bat species *My. capaccini*, which is a bat species mostly insectivorous, but also may consume some fish (vertebrates). As indicated by Ingala et al., [21], carnivorous bats had distinct pathways compared with other animal or plant-based habits. When the analysis of the taxa contributing to those specific pathways was performed in the present paper, the bat species *My. capaccinii*, despite having the highest number of specific pathways had a very low number of taxa participating in those 13 pathways. In particular, for the bat species *My. myotis* and *My. vivesi* the genus *Mycobacterium* and the species *Photobacterium damselae* had the highest contributions. Both of those taxa can cause diseases, and in particular the *P. damselae* was reported to be pathogenic for marine animals and also humans [81]. In humans, it was recorded as an opportunistic pathogen that can cause fasciitis that can even lead to death [82]. Health status of bats is not mentioned in the original paper, and further studies should be performed to correlate the health of the animals with the taxa identified.

The lower variance of microbial functional profiles compared with their taxonomic profiles and the relative functional stability of the microbiome in certain environments make predictions of average gene profiles rather reliable [76]. However, a limitation of amplicon-based functional predictions is that it varies across sample types and functional categories [76], as inferences are biased towards existing reference genomes and cannot provide resolution to distinguish strain-specific functionalities [39]. Considering that the bat microbiome is relatively poorly characterized [21], some of the identified taxa may not have reference genomes and/or close matches with the available genomes. In addition, amplicon-based functional predictions cannot distinguish between active and inactive bacterial constituents in the microbiome (see e.g., [83]), which results in the wrong assumption that all predicted pathways are active [21]. These limitations mean that rare and strain-specific functions [39], potentially present in bats microbiome with different levels of activation, may have not been detected in our study. Shotgun metagenomics sequencing and transcriptomics could reveal changes in active functional pathways related gut microbiome in response to dietary shifts in bats.

## Conclusions

Diet is one of the major determinants of the gut bacterial community and is directly influenced by the type of food consumed by the host. We found that *My. myotis* has the highest network complexity and a more abundant core microbiome among the studied species, while *My. capaccini* differed the most regarding its functional prediction, in the presence of particular protein-coding pathways. Animal-based diets can shape the gut microbiome very differently, even for bat species that generally have the same feeding type (e.g., insectivorous). Not only diet composition, but also diversity, as well host ecology predict microbial diversity, throughout host sociality, roost selection and distribution. Specific pathways are more representative in the Vespertilionidae than in the Miniopteridae family, although *Mi. schreibersii* is the richest in functional pathways (likely caused by differences in ecology). The use of network analysis may improve our understanding of the microbiome of bats, providing further clues to entangle basic differences and to evaluate the importance of different evolutionary pathways driving its development.

## Supplementary Information

**Additional file 1:** **Supplementary File 1.** Script code used to infer co-occurrence networks.

**Additional file 2:** **Supplementary Table 1.** List of shared and unique bacterial taxa in M. schreibersii, My. capaccinii, My. myotis, My. pilosus, and My. vivesi microbiome.

**Additional file 3:** **Supplementary Table 2.** List of shared and unique bacterial nodes in the M. schreibersii, My. capaccinii, My. myotis, My. pilosus, and My. vivesi co-occurrence networks.

**Additional file 4:** **Supplementary Table 3.** List of shared and unique bacterial nodes with various values of EV in the M. schreibersii, My. capaccinii, My. myotis, My. pilosus, and My. vivesi co-occurrence networks.

**Additional file 5:** **Supplementary Table 4.** List of shared and unique bacterial nodes with various values of BNC in the M. schreibersii, My. capaccinii, My. myotis, My. pilosus, and My. vivesi co-occurrence networks.

**Additional file 6:** **Supplementary Table 5.** Jaccard index for the pairwise comparisons of all five bat species considered in this study.

**Additional file 7:** **Supplementary Table 6.** Names of unique predicted pathways for three bat species.

**Additional file 8:** **Supplementary Figure 1.** Microbial co-occurrence network of different bat species. The nodes are representing bacterial taxa and the edges represent co-occurrence correlation. Node size is proportional to the eigenvector centrality (A) and BNC (B). The edges are connecting links with negative and positive interactions, respectively (SparCC > 0.60 or <-0.60).

**Additional file 9:** **Supplementary Figure 2.** Network tolerance to taxa removal for five different bat species. *Mi. schreibersii, My. capaccinii, My. myotis, My. pilosus and My vivesi* networks were subjected to direct cascading or random removal based on their BNC value. Loss of connectivity values ranges between 0 (maximum of connectivity between nodes) and 1 (total disconnection between nodes).

**Additional file 10:** **Supplementary Figure 3.** Network tolerance to taxa removal using direct (green line), cascading (red line), or random (blueline) removal based on their BNC value for all five bat species. Loss of connectivity values ranges between 0 (maximum of connectivity between nodes) and 1 (total disconnection between nodes).

**Additional file 11:** **Supplementary Figure 4.** Predicted pathways with differential abundance in different bat species. The heatmap is showing the pathways for all bat species with significant differences in their abundance (expressed as clr) (Welch's t-test, *p* < 0.05).

## Data Availability

All the datasets shown in the present study can be found at the SRA repository https://www.ncbi.nlm.nih.gov/sra (Accession numbers: ERR7141691-ERR7141699; ERR7141703-ERR7141706; ERR7141707- ERR7141719; ERR7141725-ERR7141728; ERR7141729-ERR7141741; ERR7141742-ERR7141748; ERR7142004-ERR7142009; ERR7142013-ERR7142015; ERR7159368; ERR7159373-ERR7159374).
