# Supplementary material for: Structural differences in the gut microbiome of bats using terrestrial vs. aquatic feeding resources
Source: BMC Microbiol. 2023 Apr 1;23:93. doi: 10.1186/s12866-023-02836-7 (PMC10067309; doi:10.1186/s12866-023-02836-7)
Supplement: Supplementary file 1 — Additional file 1: Supplementary File 1. Script code used to infer co-occurrence networks. [file 12866_2023_2836_MOESM1_ESM.docx]

**Supplementary File 1.** Script code used to infer co-occurrence networks.

install.packages ("openxlsx")

install.packages ("SpiecEasi")

install.packages ("igraph")

install.packages('devtools')

install_github("zdk123/SpiecEasi")

library(devtools)

library ("openxlsx")

library ("SpiecEasi")

library ("igraph")

latabla <- as.matrix(read.xlsx("Table16S.xlsx", sheet="Mschreibersii", startRow = 1, colNames = TRUE, rowNames = TRUE, detectDates = FALSE, rows = NULL, cols = NULL,

check.names = FALSE,

namedRegion = NULL, na.strings = "NA", fillMergedCells = FALSE))

# latabla <- latabla[,-ncol(latabla)]

temporal <- rowSums(latabla) ## Removing empty rows

pointer <- which(temporal>0)

latabla3 <- latabla[pointer,] ## Making a new table without the empty rows

latabla <- t(latabla3)

sparcc.latabla <- sparcc(latabla, iter=20, inner_iter=10, th=0.3)

sparcc.graph <- sparcc.latabla$Cor

## correlation cutoff of 0.75

sparcc.cutoff <- 0.75

## Obtain positive and negative interactions

sparcc.graph <- ifelse(abs(sparcc.latabla$Cor) >= sparcc.cutoff, sparcc.latabla$Cor, 0)

## Or use this for only positive interactions

#sparcc.graph[sparcc.graph<0.50] <- 0

colnames(sparcc.graph) <- colnames(latabla)

rownames(sparcc.graph) <- colnames(latabla)

diag(sparcc.graph) <- 0

# Create igraph objects and export to "graphml" for Gephi

ig.sparcc <- graph.adjacency(sparcc.graph, mode = "undirected", weighted = TRUE, diag = FALSE, add.colnames = TRUE)

write_graph(ig.sparcc, "Mschreibersii.graphml", format = c("graphml"))
