# Supplementary material for: Structural differences in the gut microbiome of bats using terrestrial vs. aquatic feeding resources
Source: BMC Microbiol. 2023 Apr 1;23:93. doi: 10.1186/s12866-023-02836-7 (PMC10067309; doi:10.1186/s12866-023-02836-7)
Supplement: Supplementary file 7 — Additional file 7: Supplementary Table 6. Names of unique predicted pathways for three bat species. [file 12866_2023_2836_MOESM7_ESM.docx]

**Supplementary Table 6.** Names of unique predicted pathways for three bat species.

| Bat species | Unique pathways |
| --- | --- |
| *My. capaccinii* | **PWY-6660** (2-heptyl-3-hydroxy-4(1H)-quinolone biosynthesis) |
|  | **BENZCOA-PWY** (anaerobic aromatic compound degradation (*Thauera aromatica*)) |
|  | **PWY-5757** (antibiotic biosynthesis) |
|  | **CENTBENZCOA-PWY** (benzoyl-CoA degradation II (anaerobic)) |
|  | **PWY-7024** (CO2 fixation) |
|  | **PWY-5743** (CO2 fixation) |
|  | **PWY-5744** (CO2 fixation) |
|  | **PWY-7401** (Superpathway: crotonate fermentation (to acetate and cyclohexane carboxylate)) |
|  | **PWY-7209** (Superpathway: pyrimidine nucleotide degradation) |
|  | **PWY-6486** (sugar acid degradation) |
|  | **PWY-5519** (sugar degradation) |
|  | **PWY-6662** (Superpathway: quinolone and alkylquinolone biosynthesis) |
|  | **PWY-5184** (Superpathway: toluene degradation) |
| *My. myotis* | **PWY-7002** (4-hydroxyacetophenone degradation) |
|  | **PWY-6919** (antibiotic biosynthesis) |
| *My. vivesi* | **PWY-7528** (Superpathway: proteinogenic amino acid biosynthesis) |
