# Supplementary figures and images for: Structural differences in the gut microbiome of bats using terrestrial vs. aquatic feeding resources
Source: BMC Microbiol. 2023 Apr 1;23:93. doi: 10.1186/s12866-023-02836-7 (PMC10067309; doi:10.1186/s12866-023-02836-7)

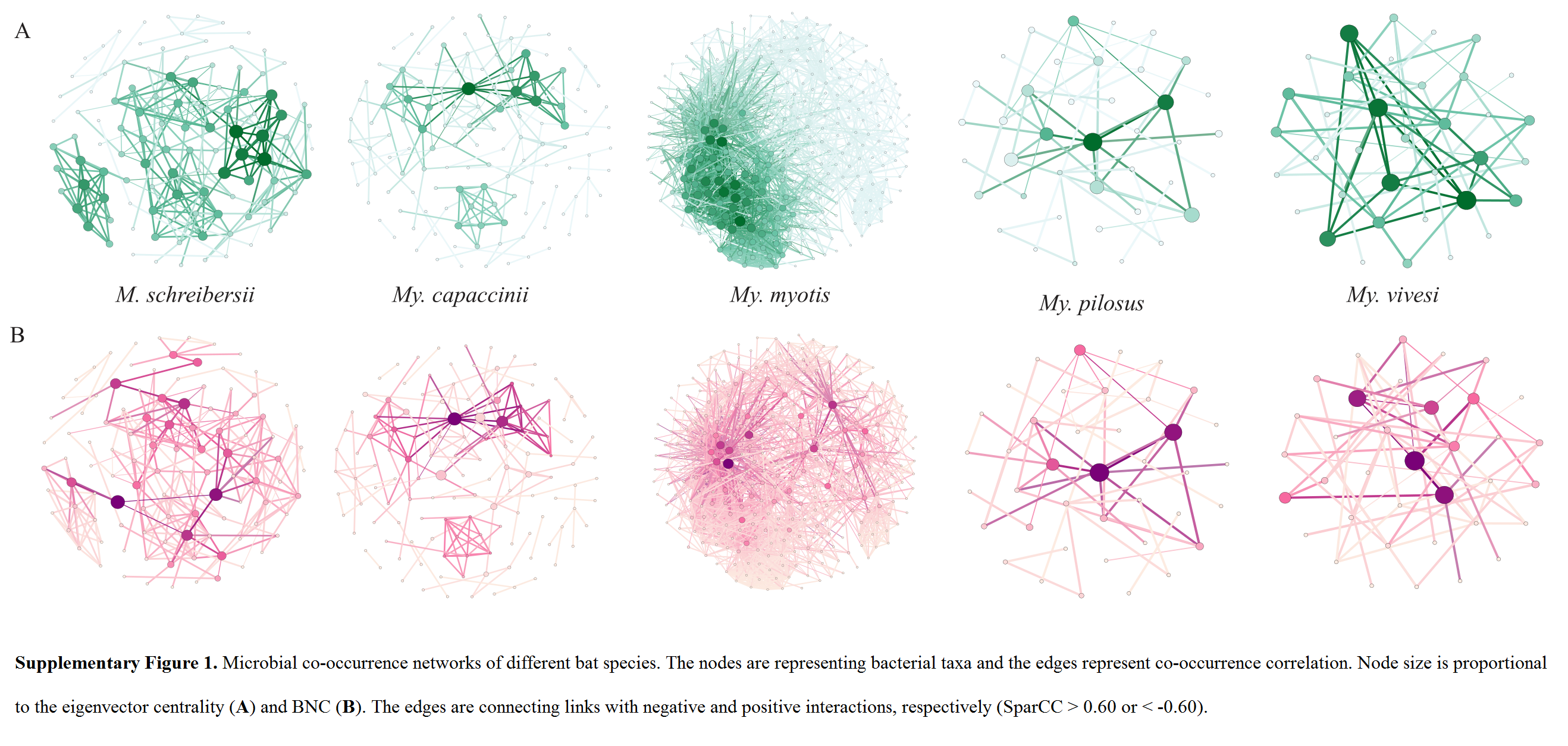

Supplement: Supplementary file 8 — Additional file 8: Supplementary Figure 1. Microbial co-occurrence network of different bat species. The nodes are representing bacterial taxa and the edges represent co-occurrence correlation. Node size is proportional to the eigenvector centrality (A) and BNC (B). The edges are connecting links with negative and positive interactions, respectively (SparCC > 0.60 or <-0.60). [file 12866_2023_2836_MOESM8_ESM.tiff]

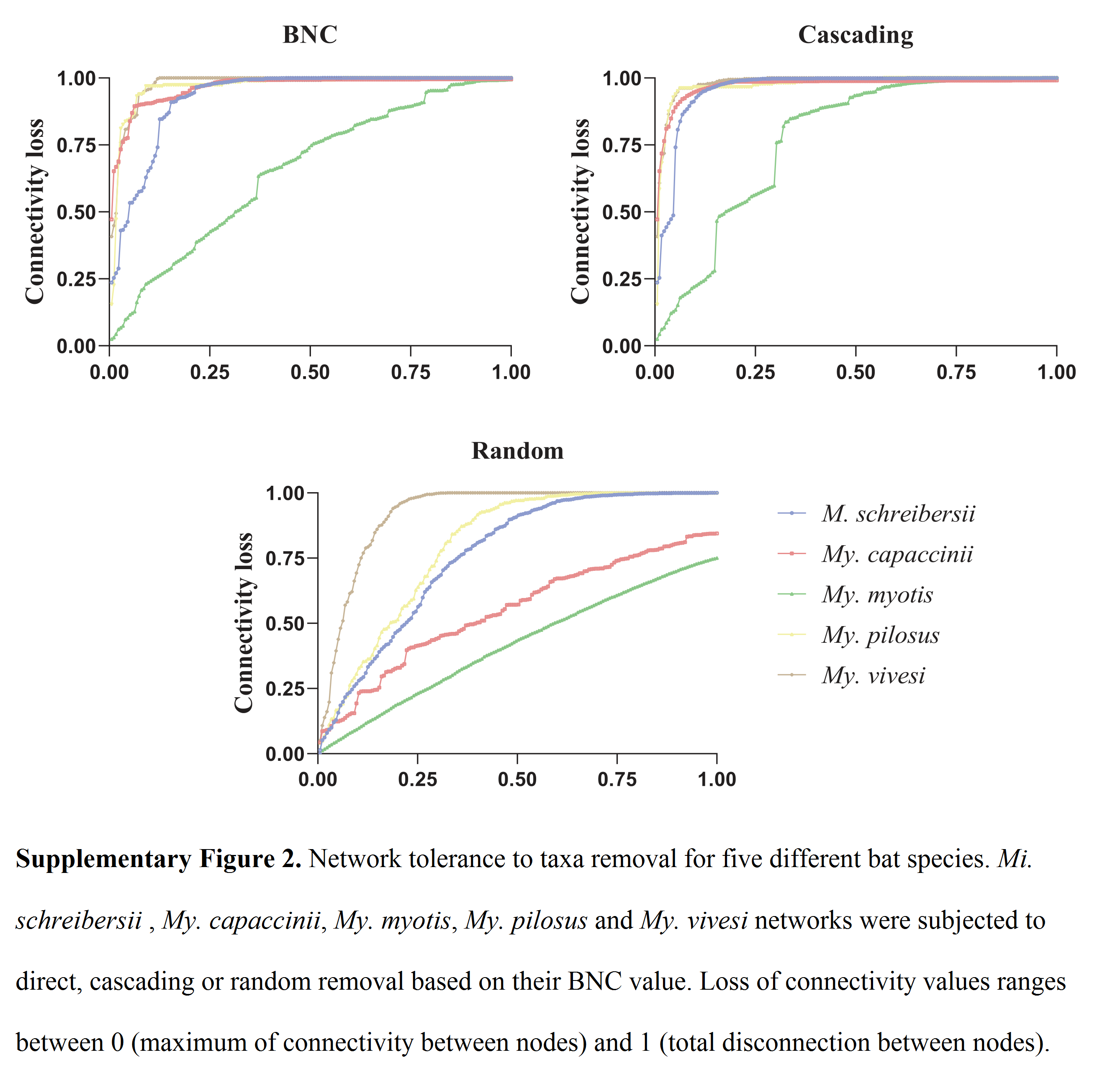

Supplement: Supplementary file 9 — Additional file 9: Supplementary Figure 2. Network tolerance to taxa removal for five different bat species. Mi. schreibersii, My. capaccinii, My. myotis, My. pilosus and My vivesi networks were subjected to direct cascading or random removal based on their BNC value. Loss of connectivity values ranges between 0 (maximum of connectivity between nodes) and 1 (total disconnection between nodes). [file 12866_2023_2836_MOESM9_ESM.tiff]

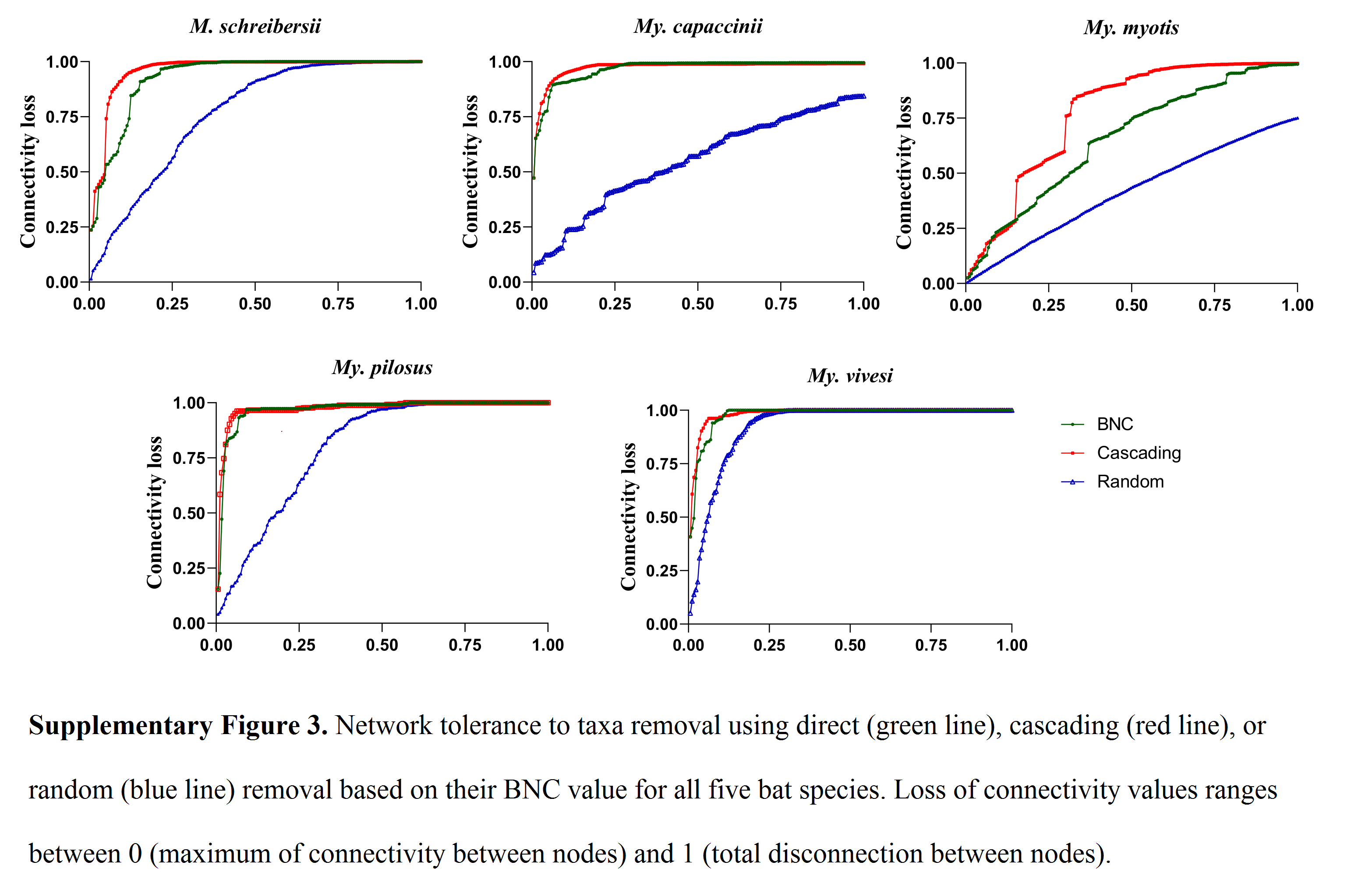

Supplement: Supplementary file 10 — Additional file 10: Supplementary Figure 3. Network tolerance to taxa removal using direct (green line), cascading (red line), or random (blueline) removal based on their BNC value for all five bat species. Loss of connectivity values ranges between 0 (maximum of connectivity between nodes) and 1 (total disconnection between nodes). [file 12866_2023_2836_MOESM10_ESM.tiff]

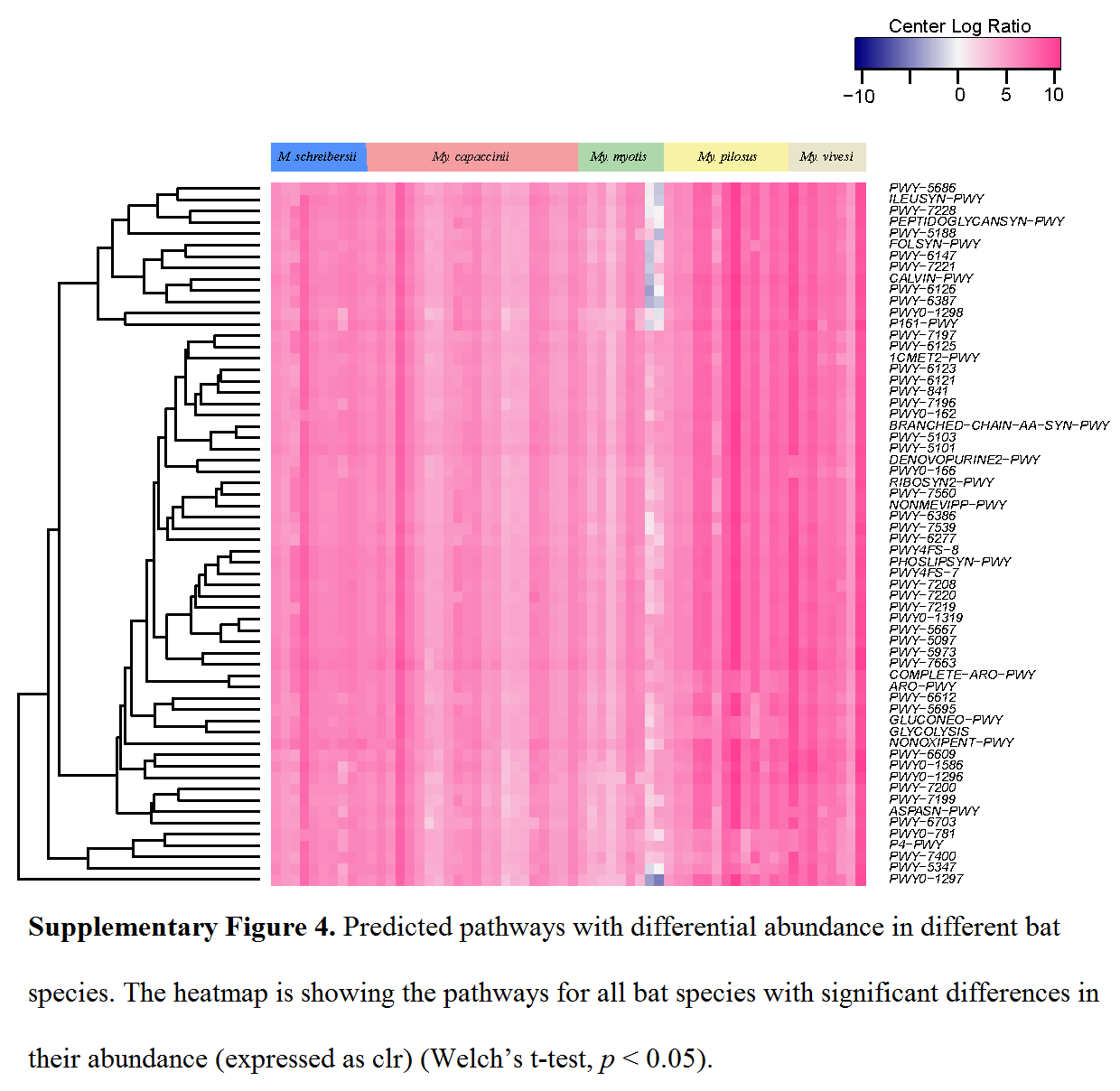

Supplement: Supplementary file 11 — Additional file 11: Supplementary Figure 4. Predicted pathways with differential abundance in different bat species. The heatmap is showing the pathways for all bat species with significant differences in their abundance (expressed as clr) (Welch's t-test, p < 0.05). [file 12866_2023_2836_MOESM11_ESM.tiff]
